# Increasing incidence of hip arthroplasty for primary osteoarthritis in 30- to 59-year-old patients: A population based study from the Finnish Arthroplasty Register

**DOI:** 10.3109/17453674.2010.548029

**Published:** 2011-02-10

**Authors:** Eerik T Skyttä, Leskinen Jarkko, Eskelinen Antti, Heini Huhtala, Remes Ville

**Affiliations:** ^1^COXA Hospital for Joint Replacement, Tampere; ^2^Centre for Rheumatic Diseases, Department of Orthopaedics, Tampere University Hospital, Tampere; ^3^Department of Orthopedics, Peijas Hospital, Helsinki University Central Hospital, Helsinki; ^4^School of Public Health, University of Tampere, Tampere, Finland

## Abstract

**Background and purpose:**

The use of hip arthroplasties is evidently increasing, but there are few published data on the incidence in young patients.

**Methods:**

We used data on total and resurfacing hip arthroplasties (THAs and RHAs) from the Finnish Arthroplasty Register and population data from Statistics Finland to analyze the incidences of THA and RHA in patients aged 30–59 years in Finland, for the period 1980 through 2007.

**Results:**

The combined incidences of THAs and RHAs among 30- to 59-year-old inhabitants increased from 9.5 per 10^5^ inhabitants in 1980 to 61 per 10^5^ inhabitants in 2007. Initially, the incidence of THA was higher in women than men, but since the mid-90s the incidences were similar. The incidence increased in all age groups studied (30–39, 40–49, and 50–59 years) but the increase was 6-fold and 36-fold higher in the latter two groups than in the first. The incidence of THA was constant; the increased incidence of overall hip arthroplasty was due to the increasing number of RHAs performed.

**Interpretation:**

We have found a steady increase in the incidence of hip arthroplasty in patients with primary hip osteoarthritis in Finland, with an accelerating trend in the past decade, due to an increase in the incidence of RHA. As the incidence of hip osteoarthritis has not increased, the indications for hip arthroplasty appear to have become broader.

75% of THAs are performed on elderly patients for painful osteoarthritis (OA); in younger patients (under 50–60 years), the proportion of OA diminishes to 42–54%. Most patients are women (Lucht 2000, Furnes et al. 2001, Puolakka et al. 2001, Malchau et al. 2002).

Several authors have reported an increasing incidence of treatment of OA with THA (Birrell et al. 1999, Ostendorf et al. 2002, Wells et al. 2002, Merx et al. 2003, Kurtz et al. 2005, 2007), but only a few authors have reported the incidences for younger patients separately (Birrell et al. 1999, Ingvarsson et al. 1999). Resurfacing hip arthroplasty (RHA) is an option marketed for younger patients, though its value is still uncertain (McGrory et al. 2009).

We examined the changes in incidence of primary THA and RHA in young patients with OA in Finland between the years 1980 and 2007.

## Patients and methods

### Inclusion criteria

All THAs and RHAs performed in patients aged 30–59 years with primary hip osteoarthritis who had been entered in the Finnish Arthroplasty Register between 1980 and 2007 were included (Table). Data concerning the Finnish population during the study period were obtained from Statistics Finland; we used the end-of-year population as an approximation of the population each year. The population data were divided into cohorts by age (30–39, 40–49, and 50–59 years) and subgroups according to sex.

**Table:** Hip arthroplasties in young patients (30–59 years of age) during 1980–2007, according to type of hip arthroplasty, sex, and age group

	n	%
Patients aged 30–59 years (primary OA)	17,248	100
THA	15,014	87
RHA	2,234	13
Proportion of women (< 60 years)	8,159	47
THA	7,335	43
RHA	824	4.8
Age groups of the study population	17,248	100
30–39 years of age	407	2.4
THA	302	1.8
RHA	105	0.6
40–49 years of age	2,619	15
THA	2,083	12
RHA	536	3.1
50–59 years of age	14,222	82
THA	12,629	73
RHA	1,593	9.2

THA: total hip arthroplasty; RHA: resurfacing hip arthroplasty.

### Incidence of hip arthroplasty

The general incidence is presented as the number of operations performed per 10^5^ person years in Finnish inhabitants aged 30–59 years. Incidence by sex is presented as the number of operations performed per 10^5^ person years in men or women aged 30–59 years. The incidences by age group are presented as the number of operations performed per 10^5^ person years in Finnish inhabitants aged 30–39 years, 40–49 years, and 50–59 years.

All incidence calculations were done separately for THAs and RHAs.

### Statistics

We analyzed the differences between every group for general incidence of THA and RHA, sex-specific incidence, and incidence by age group by calculating incidence rate ratio (IRR) using Poisson regression. For each comparison, the IRR for annual increase in incidence was calculated first and the effect of the group was then calculated. The increase in IRR is presented as a percentage (e.g. IRR = 1.048 is shown as 4.8%) with 95% CI. The differences were considered to be statistically significant if p-values were less than 0.05 in a two-tailed test. We used STATA 8.2 statistical software (StataCorp, College Station, TX).

## Results

### Patient characteristics

Between 1980 and 2007, 17,248 hip arthroplasties were performed for primary osteoarthritis in patients aged less than 60 years in Finland (Table). Of these operations, 8,159 (47%) were performed on women and 2,234 (13%) were RHAs. All RHAs were performed from 2001 through 2007. Median age at the time of surgery was 55 years (interquartile range: 51–58).

### General incidence

**Figure 1.**
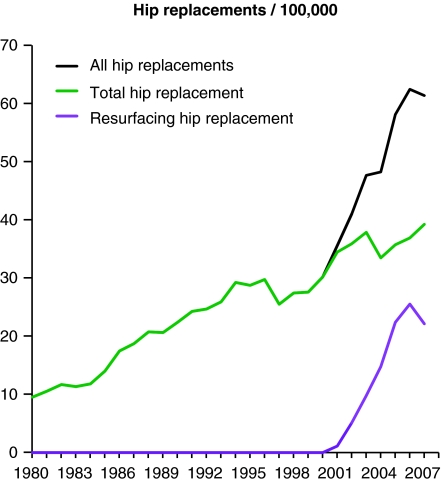
General incidence of hip arthroplasty for primary osteoarthritis in young patients (30–59 years of age).

There was a 6-fold increase in the incidence of hip arthroplasty over the whole study period: the incidence increased from 9.5 operations per 10^5^ inhabitants in 1980 (in patients aged 30–59 years) to 61 in 2007 (Figure 1). The peak incidence was noted in 2006 (62 per 10^5^) but the decrease in the number of RHAs reduced the general incidence despite the steady growth in the number of THAs. The IRR for annual increase in general incidence was 6.6% (CI: 6.3–6.8).

The incidence of THA grew steadily from 9.5 to 39 per 10^5^ during the period 1980–2007, with 858 THAs performed in 2007. The IRR for annual increase in incidence of THA was 4.5% (CI: 4.3–4.7). The incidence of RHA increased rapidly from 1.1 to 26 operations per 10^5^ during 2001–2006 (Figure 1), but the incidence decreased to 22 in 2007, with 484 RHAs performed in that year. The IRR for annual increase in incidence of RHA was 37% (CI: 36–40).

### Incidence by sex

**Figure 2.**
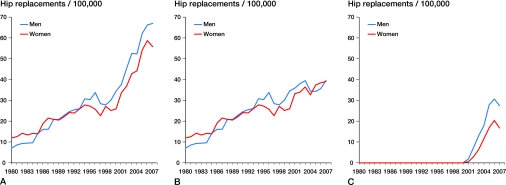
A. Incidence of all hip arthroplasties for primary osteoarthritis in young patients (30–59 years of age), by sex. The increase in incidence during 1980–2007 was greater in men than in women (p < 0.001). B. Incidence of total hip arthroplasty (THA) for primary osteoarthritis in young patients (30–59 years of age), by sex. C. Incidence of resurfacing hip arthroplasty (RHA) for primary osteoarthritis in young patients (30–59 years of age), by sex. The increase in incidence during 2001–2007 was greater in men than in women (p < 0.001).

In 1980, hip arthroplasties were performed 1.7 times more frequently in women than in men (11.9 vs. 7.1 per 10^5^ inhabitants) (Figure 2A). However, regarding whether there was any difference between the sexes, the IRR for increase in incidence of hip arthroplasty was 9.1% (CI: 5.9–12.4) higher in men than in women (p < 0.001). Given the IRR for annual increase in general incidence of 6.6% (see above), hip arthroplasties were performed 1.2 times more frequently in men than in women (67 vs. 56 per 10^5^) in 2007.

There were no statistically significant differences in annual increase in the incidence of THA between men and women (IRR = 3.0%, CI: –1.0 to 5.9; p = 0.1) (Figure 2B). In contrast, the IRR for increase in incidence of RHA was 68% (CI: 54–83) higher in men than in women (p < 0.001) (Figure 2C).

### Incidence by age group

**Figure 3.**
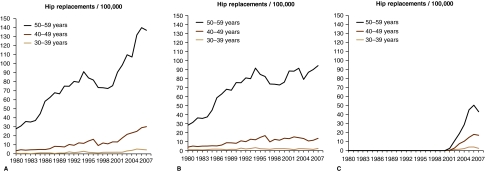
A. Incidence of all hip arthroplasties for primary osteoarthritis in young patients (30–59 years of age), by age group. B. Incidence of total hip arthroplasty (THA) for primary osteoarthritis in young patients (30–59 years of age), by age group. C. Incidence of resurfacing hip arthroplasty (RHA) for primary osteoarthritis in young patients (30–59 years of age), by age group.

In all age groups, there appeared to be an increase in incidence of all hip arthroplasties—THAs and RHAs (Figure 3A). In the 30–39-year age group, the IRR for annual increase in incidence of hip arthroplasty was 5.2% (CI: 5.0–5.4). Between groups, the difference in IRR for increase in incidence of hip arthroplasty was 6-fold (614%, CI: 594–634; p < 0.001), i.e. any given incidence in the 30–39-year group was 6-fold higher in the 40–49-year group, and it was 36-fold higher in the 50–59-year group.

In THAs, the difference between groups was even more pronounced, with a 7-fold increase in IRR for increase in incidence of hip arthroplasty (690%, CI: 665–716; p < 0.001). The IRR for annual increase in incidence of THA was 3.2% (CI: 2.9–3.4). In RHAs, there was also a marked 3-fold increase in IRR between groups (320%, CI: 298–343, p < 0.001).

## Discussion

Our main finding was the steady annual increase in incidence of hip arthroplasty in young patients over the period 1980–2007, with an accelerating trend over the past decade (Figure 1). The incidence of THA had a constant annual increase of 4.5%, and the acceleration appeared to be triggered by RHA quickly becoming popular since its inception in 2001. In Finland, the overall hip arthroplasty (THA and RHA) incidences have grown more quickly in men than in women. In the age groups 30–39, 40–49, and 50–59 years, the increase in incidence has been more pronounced in the latter 2 groups, the most substantial growth being in the last group.

Registry-based studies have certain limitations. The Finnish Arthroplasty Register covered 90% of hip implants in the year 1995, and after that the coverage increased to 98% (Mäkelä et al. 2008). This might explain a minor part of the increase shown in our study. Because of the accurate civil registry of Statistics Finland and the low level of illegal immigration in Finland, the population data can be assumed to correspond to the actual population. Nevertheless, limitations in the accuracy of the data and in conclusions drawn may result from inconsistencies or errors in the diagnostic coding entered into the registry (Ingvarsson et al. 1999, Pedersen et al. 2004)

There have been few studies in which separate incidences have been reported for young patients. Kurtz et al. (2005) found a 50% increase in incidence in THA in the USA during 1990–2002, and, although not directly stated, an approximate annual increase in incidence of 4.5% in the youngest-studied group (45–64 years) can be calculated from their article, which coincides with our results. In a study from NHS hospitals in the UK in 1996 (Dixon et al. 2004), incidences for THA in women in the age groups < 39, 40–49, and 50–59 years were 2.1, 16, and 115 per 10^5^ individuals. In men, the corresponding incidences were 1.4, 13, and 90 per 10^5^. In our study, the incidence of THA in women was higher than in men only until the mid-1990s. Presently, the incidence of THA is similar in men and women, and the incidence of all hip arthroplasties is 20% higher in men (Figure 2). Another study using the same UK NHS hospital data from 1996 showed total incidence rates of 12 and 189 per 10^5^ for the age groups 0–54 and 55–64 years (Birrell et al. 1999). The authors also projected the need for THA in the UK—but only for patients aged over 55 years—using estimated changes in demographic data until 2026. The projected overall increase in number of THAs was 24% for the period 1996–2006 in the youngest group of 55–64-year-old patients with OA. During the same time period in our study, the increase in number of hip arthroplasties in the oldest group (50–59 years) was 42% and 121% for THAs and all hip arthroplasties, respectively. However, any direct comparison between these studies is impossible, because the age groups were not the same, registration differed considerably, and demographic changes (immigration, birth rates, etc) can be divergent. We cautiously conclude that our increasing incidence rates support the direction of the projected changes in hip arthroplasty numbers in the study by Birrell et al. (1999).

Wells et al. (2002) reported an increase in the incidence of THA from 48 to 84 per 10^5^ in patients with primary osteoarthritis in the province of South Australia between 1988 and 1998. Between 1994 and 1998 in the total Australian population, the incidence rates increased from 51 to 61 per 10^5^. The article does not provide actual incidence rates in numbers for different age groups but the annual increase in incidence of THA was 5% in all age groups (Wells et al. 2002).

We have shown an obvious increase in the incidence of hip arthoplasty in young patients. The finding is apparent in both sexes and even in the youngest age groups. Demographic changes alone give no explanation to the changes in incidence. Furthermore, the prevalence of hip OA and hip symptoms has remained unchanged in Finland and other countries over earlier decades (Danielsson and Lindberg 1997, Heliövaara et al. 2007). Orthopedic surgeons are ultimately responsible for the increased rate, as they do the operations. Have we changed our approach due to pressure from patients, industry, or new clinical data? Our modern way of life possibly requires higher functional ability in both work and leisure. Younger patients may opt for elective operations at an earlier stage, with milder symptoms, than previously. Prosthesis manufacturers are eager to develop and market new concepts and designs, including novel THA bearings and RHA. This marketing takes place both indirectly through community- or internet-based advertising, or directly through their relationships with orthopedic surgeons. Good long-term results in terms of implant survivorship of THA in older age groups have encouraged surgeons to broaden the indications for THA to younger and more active patients. Whatever the underlying reasons for the observed increase in the incidence of hip arthroplasty, the advent of RHA has had a substantial impact on the incidence of hip arthroplasty.

In conclusion, age- and sex-standardized incidences of THA and RHA in young patients with primary osteoarthritis have increased in Finland. This phenomenon has been especially strong during the current decade. There is no single explanatory factor for this growth. Some of the increase in incidence may be explained by hospital volume or geographic location of the patients. In general, the observed growth is so rapid and high, that given the lack of information on long-term outcome of hip arthroplasties in younger patients and the recent concerns about metal-on-metal hip arthroplasties (Langton et al. 2010), we should really be worried about a rapid increase in revision hip arthroplasties in the near future. Future long-term results of hip arthroplasty in younger age groups will reveal whether or not our current policy of widespread use of hip arthroplasty in younger patients is wise.
